# Organizing integrated health-care services to meet older people’s needs

**DOI:** 10.2471/BLT.16.187617

**Published:** 2017-05-26

**Authors:** Islene Araujo de Carvalho, JoAnne Epping-Jordan, Anne Margriet Pot, Edward Kelley, Nuria Toro, Jotheeswaran A Thiyagarajan, John R Beard

**Affiliations:** a Department of Ageing and Life Course, World Health Organization, 20 avenue Appia, 1211 Geneva 27, Switzerland.; b Independent consultant, Seattle, United States of America.; c Department of Evidence, Research, Action on Mental and Brain Disorders, World Health Organization, Geneva, Switzerland.; d Service Delivery and Safety Department, World Health Organization, Geneva, Switzerland.; e Department of Services Organization and Clinical Interventions, World Health Organization, Geneva, Switzerland.

## Abstract

In most countries, a fundamental shift in the focus of clinical care for older people is needed. Instead of trying to manage numerous diseases and symptoms in a disjointed fashion, the emphasis should be on interventions that optimize older people’s physical and mental capacities over their life course and that enable them to do the things they value. This, in turn, requires a change in the way services are organized: there should be more integration within the health system and between health and social services. Existing organizational structures do not have to merge; rather, a wide array of service providers must work together in a more coordinated fashion. The evidence suggests that integrated health and social care for older people contributes to better health outcomes at a cost equivalent to usual care, thereby giving a better return on investment than more familiar ways of working. Moreover, older people can participate in, and contribute to, society for longer. Integration at the level of clinical care is especially important: older people should undergo comprehensive assessments with the goal of optimizing functional ability and care plans should be shared among all providers. At the health system level, integrated care requires: (i) supportive policy, plans and regulatory frameworks; (ii) workforce development; (iii) investment in information and communication technologies; and (iv) the use of pooled budgets, bundled payments and contractual incentives. However, action can be taken at all levels of health care from front-line providers through to senior leaders – everyone has a role to play.

## Résumé

Dans la plupart des pays, un changement fondamental de priorité dans l'organisation des soins cliniques destinés aux personnes âgées est nécessaire. Plutôt que d'essayer de gérer la variété des maladies et symptômes de manière individuelle, l'accent devrait être mis sur les interventions qui optimisent les capacités physiques et mentales des personnes âgées sur tout leur parcours de vie et qui leur permettent de continuer de réaliser les activités qui comptent pour elles. Mais cela suppose de modifier le mode d'organisation des prestations, avec une meilleure intégration à l'intérieur du système de santé et entre les services de santé et d'aide sociale. Cela ne signifie pas que les structures existantes doivent fusionner, mais plutôt qu'une grande diversité de prestataires doit travailler ensemble de manière plus coordonnée. Des données factuelles montrent que des prestations de santé et d'aide sociale intégrées entraînent de meilleurs résultats sur la santé des personnes âgées que les prestations de soins habituelles, pour un coût équivalent; d'où l'obtention d'une meilleure rentabilité des investissements comparativement aux modes de travail classiques. Elles permettent aussi aux personnes âgées de s'impliquer socialement et d'apporter leurs contributions à la société pendant plus longtemps. Une telle intégration est particulièrement importante au niveau des soins cliniques: des évaluations exhaustives devraient être réalisées chez les personnes âgées dans une optique d'optimisation de leurs capacités fonctionnelles, et les plans de soins devraient être communs à tous les prestataires. Au niveau du système de santé, l'intégration des prestations nécessite: (i) l'adoption de politiques, programmes et cadres réglementaires favorables; (ii) le développement du personnel de santé; (iii) un investissement dans les technologies de l'information et de la communication; et (iv) la mise en place de budgets communs, de paiements regroupés et de mesures contractuelles incitatives. Toutefois, des actions peuvent être entreprises à tous les niveaux d'organisation des soins de santé, depuis les prestataires de première ligne jusqu'aux hauts responsables – tout le monde a un rôle à jouer.

## Resumen

En la mayoría de países se necesita un cambio fundamental en el enfoque de la atención clínica que reciben las personas mayores. En lugar de intentar gestionar numerosas enfermedades y síntomas por separado, debería ponerse énfasis en las intervenciones que optimizan las capacidades físicas y mentales de las personas mayores durante su vida y que les permitan hacer lo que ellos valoran. Esto, a su vez, requiere un cambio en la forma en la que se organizan los servicios: debería haber más integración dentro del sistema sanitario y entre los servicios sanitarios y sociales. Las estructuras organizativas existentes no deben fusionarse, sino que el amplio conjunto de proveedores de servicios debe trabajar conjuntamente de una forma más coordinada. Las pruebas indican que la atención sanitaria y social integrada para las personas mayores contribuye a unos mejores resultados sanitarios a un coste equivalente a la atención habitual. De esta forma, se obtiene una mayor rentabilidad de la inversión que la obtenida con formas de trabajar más familiares. Además, las personas mayores pueden participar y contribuir en la sociedad durante más tiempo. La integración a nivel de la atención clínica es especialmente importante: las personas mayores deberían someterse a asesoramiento integral con el objetivo de optimizar la capacidad funcional, y deberían compartirse los planes de atención entre todos los proveedores. A nivel del sistema sanitario, la atención integrada requiere: (i) política, planes y marcos normativos de apoyo; (ii) desarrollo del personal sanitario; (iii) inversión en tecnologías de la información y comunicación; y (iv) el uso de presupuestos y pagos combinados e incentivos contractuales. No obstante, esto puede realizarse en todos los niveles de la atención sanitaria, desde los proveedores de primera línea hasta el personal directivo; todos juegan un papel.

## ملخص

في معظم البلدان، توجد حاجة إلى تحول رئيسي في تركيز الرعاية السريرية لكبار السن. فبدلاً من محاولة إدارة العديد من الأمراض والأعراض بطريقة مفككة، ينبغي التركيز على التدخلات التي تحسن القدرات البدنية والعقلية لكبار السن على مدى حياتهم بالطبع والتي تمكنهم من القيام بالأشياء التي يُقدروها. وهذا بدوره يتطلب تغييرًا في طريقة تنظيم الخدمات: ينبغي أن يكون هناك تكامل أكبر داخل النظام الصحي وبين الخدمات الصحية والاجتماعية. ولا يلزم دمج الهياكل التنظيمية القائمة؛ بل يجب أن تعمل مجموعة كبيرة من مقدمي الخدمات معًا بطريقة أكثر تنسيقًا. وتشير الدلائل إلى أن الرعاية الصحية والاجتماعية المتكاملة لكبار السن تسهم في تحقيق نتائج صحية أفضل بتكلفة تعادل الرعاية المعتادة، مما يعطي عائد استثمار أفضل من الطرق المألوفة للعمل. وعلاوة على ذلك، يمكن لكبار السن المشاركة في المجتمع والمساهمة فيه لفترة أطول. للتكامل في مستوى الرعاية السريرية أهمية خاصة: حيث ينبغي أن يخضع كبار السن لتقييمات شاملة بهدف تحسين القدرة الوظيفية، وينبغي مشاركة خطط الرعاية بين جميع مقدمي الخدمات. وعلى صعيد النظام الصحي، تتطلب الرعاية المتكاملة ما يلي: (أ) السياسات والخطط والأطر التنظيمية الداعمة؛ و(ب) تنمية القوى العاملة؛ و(جـ) الاستثمار في تكنولوجيات المعلومات والاتصالات؛ و(د) استخدام الميزانيات المجمعة والمدفوعات المجمعة والحوافز التعاقدية. ومع ذلك، يمكن اتخاذ إجراءات على جميع مستويات الرعاية الصحية بدءًا من مقدمي الرعاية في الخطوط الأمامية إلى كبار المسؤولين – فكل شخص دورٌ للقيام به.

## 摘要

许多国家需对老年人临床护理的重点进行根本性转变。 重点应放在能够在生活过程中优化老年人的行为及心智能力并使其能够做其认为有价值的事情的干预措施上，而不是试图以一种杂乱的方式应付无数的疾病和症状。 相应地，这要求改变建立服务的方式： 应加强卫生系统内部及卫生与社会服务之间的一体化。 现有组织机构无需进行合并，但是，各类服务提供商必须以更协调的方式进行合作。 证据表明对于老年人来说，在与常规护理成本相同的情况下，一体化的健康与社会护理更利于产生更好的健康结果，因此较更多常见工作方式而言，能带来更大的投资回报。 此外，老年人能够更长久地参与社会并为社会做出贡献。 临床护理级别的一体化尤为重要： 老年人应在优化功能性能力的目标下接受全面评估，并且护理计划应与所有供应商共享。 卫生系统级别的一体化护理需要： (i) 支持性政策、计划及监管框架；(ii) 人力开发；(iii) 投资于信息与通讯技术；以及 (iv) 使用集合预算、捆绑支付和契约式激励。 但，从健康护理的一线提供商到高级领导者，所有层面均可采取行动——各方均发挥自己的作用。

## Резюме

В большинстве стран необходим фундаментальный сдвиг в сфере оказания клинической помощи пожилым людям. Вместо того чтобы пытаться лечить бесчисленные болезни и симптомы, акцент должен делаться на вмешательствах, которые оптимизируют физические и умственные способности пожилых людей в течение их жизни и позволяют им быть более дееспособными. Это, в свою очередь, требует изменения порядка организации услуг, а именно большей интеграции в систему здравоохранения, а также лучшего взаимодействия между здравоохранением и социальными услугами. Не должно происходить слияния существующих организационных структур. Скорее, широкий круг организаций, предоставляющих услуги по оказанию медицинской и социальной помощи, должен работать сообща более согласованным образом. Имеющиеся данные свидетельствуют о том, что комплексная система здравоохранения и социальной помощи пожилым людям способствует улучшению клинических последствий при стоимости, эквивалентной стандартной медицинской помощи, что дает лучшую отдачу от инвестиций, чем более привычные методы работы. Более того, пожилые люди более продолжительное время могут принимать участие в общественной жизни и вносить в нее свой вклад. Интеграция на уровне клинической помощи особенно важна: пожилые люди должны проходить всестороннее обследование с целью оптимизации умственных и физических способностей, а планы по уходу должны распределяться между всеми организациями, предоставляющими услуги по оказанию медицинской и социальной помощи. На уровне системы здравоохранения комплексный уход требует: (I) применения поддерживающей политики, планов и нормативной базы; (II) подготовки трудовых ресурсов; (III) инвестиций в информационно-коммуникационные технологии; (IV) использования объединенных бюджетов, комплексной оплаты и вознаграждений, предусмотренных контрактами. Тем не менее действия могут быть предприняты на всех уровнях здравоохранения: от врачей, работающих непосредственно с пациентами, до старших руководителей. Каждый должен играть определенную роль.

## Introduction

In 2015, the World Health Organization (WHO) published the first *World report on ageing and health*.^1^ This was followed in 2016 by the World Health Assembly’s adoption of a *Global strategy and plan of action on ageing and health*,^2^ which provide a clear mandate for action across health and social care sectors. Both documents reflect a new conceptual model of healthy ageing that is built around the functional ability of older people to do the things they value, rather than around the absence of disease. At the same time, through the United Nations, Member States adopted the 2030 Agenda for Sustainable Development,^3^ pledging that no one will be left behind and that every human being will have an equal opportunity to fulfil their potential with dignity.

These documents call for major reforms to health and long-term care systems and for a fundamental change in the focus of clinical care for older people. Instead of trying to manage an array of diseases and treat specific symptoms in a disjointed fashion, the *World report on ageing and health*^1^ proposes prioritizing interventions that optimize older people’s physical and mental capacities over their life course. This, in turn, requires a change in the way health and social services are organized: there should be more integration within the health system and between health and social-care services. In this paper we discuss WHO’s approach to integrated health care for older people. However, the integration of health and long-term care systems is beyond its scope.

## Importance of integrated health care

As people age, their health issues tend to become more chronic and complex, and multimorbidity – that is the presence of multiple chronic conditions at the same time – becomes the norm rather than the exception. Physical, sensory and cognitive impairments become more prevalent and older people can develop complex health states, such as frailty, urinary incontinence and an increased risk of falling. These health states cannot be placed in discrete disease categories. The risk of having multiple noncommunicable health conditions also increases with age and, if not properly addressed through robust care coordination, these conditions can lead to polypharmacy, hospitalization and death.

Providing care for older people with multiple health issues is commensurately complex. Numerous health workers may be involved with a single person’s care, especially in countries where medical specialists are widely available. Yet many existing health systems manage health issues in a disconnected and fragmented way and there is a lack of coordination across care providers and settings and in the timing of the care provided. In one survey of older adults in 11 high-income countries, up to 41% reported problems with the coordination of care over the past two years.^4^ Such fragmentation can result in health care that not only fails to adequately meet the needs of older people but also leads to substantial, avoidable costs, both for older people and for the health-care system.^5^

Older people often find it difficult to use health services even when they are available. In low- and lower-middle-income countries, older people use health services significantly less frequently than younger people despite suffering poorer health.^6^^,^^7^ The greatest barriers to use appear to be the cost of health-care visits and difficulties with transportation: more than 60% of older people in low-income countries did not access health care because of the cost of the visit, the absence of transportation or an inability to pay for transportation.^1^ Older people living in rural areas may have particular problems with transportation because care services are often concentrated in large cities far from their homes and communities. Consequently, multisectoral action involving the transportation sector in addition to social care is neeed.^5^^,^^8^ In addition, health services could be located closer to where older people live.

Other widespread barriers to access arise from the failure of health services to take into account the limitations in physical capacity common in older people. There may be a lack of accessible toilets, long queues, physical barriers to access and communication barriers resulting from inadequate provision of information for people with hearing loss or visual impairment. Long waiting times and queues can be particularly challenging for older people with physical disabilities, restricted mobility or urinary incontinence.^9^^,^^10^ Another important factor that discourages older people from seeking care or results in their disengagement from health services is the ageist attitudes that are widespread in many societies, even among health professionals.^11^

The ubiquity of these barriers means that, in addition to improving the coordination of care, integrated health-care services must be tailored to the specific needs of older people – services should be provided without discrimination by age, close to where people live and within an infrastructure that is friendly to older people. The evidence suggests that integrated health care contributes to better health outcomes in older people at a cost equivalent to usual care.^12^^–^^15^ Consequently, integrated health care gives a better return on investment than more familiar ways of working and helps older people to continue participating in, and contributing to, society.

## Reorganizing health services

Delivering age-friendly, integrated health-care services will require a transformation in the way health systems are designed. Services will have to be oriented around the needs of older people rather than around the needs of the services themselves. And, they will have to serve older people with a high and stable level of intrinsic capacity, those with a declining capacity and those whose capacity has deteriorated to the point where they require the care and support of others. Intrinsic capacity is defined by WHO as the combination of the individual’s physical and mental (including psychosocial) capacities.^1^ Functional ability, in contrast, relates to the combination and interaction of an individual’s intrinsic capacity and the characteristics of the environment he or she inhabits.

A shift in the type of care older people receive is also needed: away from a singular focus on the management of specific diseases and conditions and towards care that aims to optimize older people’s intrinsic capacity over their life course.^12^^–^^14^ The aim is not to devalue good disease management but is rather to emphasize that an older person’s physical and mental capacity should be the focus of, and the starting point for, coordinated health interventions.

Many people reach a point in older age when they are no longer able to perform the basic tasks of day-to-day life without assistance. They reach the stage of care-dependency, which is primarily addressed through long-term care systems. Nonetheless, health-care systems are still important for people with a serious loss of capacity – these people may require ongoing disease management, rehabilitation, or palliative and end-of-life care. In addition, health-care systems must ensure timely access to primary, specialty and acute care when needed. Evidence exists that specialist, acute, geriatric wards can deliver higher-quality care with shorter hospital stays and lower costs.^15^^–^^17^

## WHO’s approach

In the *World report on ageing and health*, WHO described the type of health care needed for ageing populations as “integrated health care for older people.”^1^ Care can be integrated at: (i) the macro level (i.e. at the policy or sector level); (ii) the meso level (i.e. at the organizational or professional level); or (iii) the micro level (i.e. at the clinical or interventional level).^18^^,^^19^ Although WHO’s approach to integrated health care for older populations spans all these levels, it emphasizes integration at the level of clinical care. In addition, it is also older person-centred, which means it adopts the perspective that older people are more than vessels for their health disorders and conditions; they are instead individuals with unique experiences, needs and preferences. People are also seen in the context of their daily lives, as part of a family and a community. Moreover, instead of being confronted by ageist attitudes, older people should have their dignity and autonomy respected and embraced in a culture of shared decision-making.

The key elements of WHO’s proposed approach and their implications for health-care systems are described in Box 1 in accordance with WHO’s health system building blocks.^1^^,^^20^ Integration at the level of clinical care is especially important for older people: they should undergo comprehensive assessments with the goal of optimizing functional ability and care plans should be shared among all care providers. Integrated health care for older people can best be achieved by bringing services together around their needs using a person-centred approach and there should be a strong emphasis on case management, support for self-management and ageing in place (i.e. community-based care).^21^ This approach is supported by observational studies and systematic reviews, though investigations were often limited in terms of the scope of the intervention, the outcomes assessed or the population studied and they were usually conducted in high-income countries (Box 2).

Box 1 **Key elements of WHO’s approach to integrated health care for older people, 2015^1^**

**Goal of integrated health care**

All elements of integrated care for older people should be based on the individual’s unique needs and preferences.

**Micro-level integration**

**Clinical care**

Integration at the clinical care level is especially important for older people and should include: (i) comprehensive assessment; (ii) a common treatment or care goal based on the individual’s intrinsic capacity and functional ability; and (iii) a care plan that is shared among all care providers.

**Meso- and macro-level integration**

**Service delivery**

Important aspects of service delivery for older people include: (i) active case-finding and management; (ii) community-based care; and (iii) home-based interventions. In addition, service delivery must be anchored to a strong and well-performing primary health-care system. Support for self-management provides older people with the information, skills and tools they need to manage their health conditions, prevent complications, maximize their intrinsic capacity and maintain their quality of life. Community engagement enables existing resources to be employed and helps provide support for older people and their families.

**Health workforce**

Health-care workers require several key competencies to provide good-quality care for older people. Training reforms are necessary to ensure they have these skills. In addition, a critical mass of specialist geriatric expertise is needed for more difficult and complex cases. Moreover, health-care workers should be deployed in a manner consistent with the objective of providing person-centred, integrated care for older people – for this purpose, multidisciplinary teams are essential. In some contexts, care coordinators and self-management counsellors might be needed.

**Information and data**

Electronic health records and shared data platforms can capture, organize and share information about individuals and clinical populations. This information can help identify older people’s needs, plan care over time, monitor responses to treatment and assess health outcomes. Information systems can also facilitate collaboration between different health-care workers and between health-care teams and their patients, who may be located in a range of settings or geographic locations. Standard assessment measures should be reviewed to ensure they are assessing outcomes important to older people, namely intrinsic capacity and functional ability.

**Health-care infrastructure, products and technology and vaccines**

The physical infrastructure of health centres and hospitals should be designed in an older age-friendly manner. In addition, older people should have access to essential medicines and to assistive and medical devices that will enable them to remain healthy, active and independent as long as possible.

**Financing**

Policy on health financing should be aligned with the goals of universal health coverage for ageing populations, which is defined by WHO as all people having access to the health services needed without risking financial hardship by accessing them. Joint funding across health and social care sectors would help ensure coordination and efficiency and is particularly important for ageing populations.

WHO: World Health Organization.

Box 2 **Evidence supporting WHO’s approach to integrated health care for older people, 1991–2015**

**Focus on intrinsic capacity^a^**

Focusing on intrinsic capacity is more effective than prioritizing the management of specific chronic diseases.^12^^–^^14^ It helps avoid unnecessary treatment, polypharmacy and their side-effects.^8^^,^^22^^,^^23^

**Comprehensive assessments and care plans**

Comprehensive assessments and care plans harmonize clinical management across different care providers and unite providers around a common goal.^24^ For people admitted to hospital, these assessments and plans can minimise the potential risk and harms of hospitalization and can facilitate successful discharge home.^16^ For people discharged to long-term care, these assessments and plans can facilitate follow-up and provide an essential link between health and social care.^25^

**Case management**

Systematic reviews have reported that case management improves intrinsic capacity, various aspects of medication management and the use of community services.^13^ Case management also improves health outcomes in older people and has clinical benefits for people with several chronic illnesses.^26^

**Support for self-management**

Structured self-management programmes have been shown to improve a wide range of outcomes in older adults. Improvements have been observed in physical activity,^27^^–^^29^ self-care,^27^ chronic pain^30^ and self-efficacy.^27^^–^^30^

**Home-based interventions**

Home visits by health professionals in the context of community-based programmes have been shown to have positive effects. A review of 64 randomized trials found that home visits were effective when they included multidimensional assessments and were carried out five or more times: the greatest overall effects were reductions in emergency department visits, hospital admissions, the length of hospital stay and the number of falls, and better physical functioning.^31^ To be as effective as possible, home-based services must be complemented by strong links to primary health-care services, must include scheduled follow-ups and must be restricted to people at a low risk of death.^32^

WHO: World Health Organization.^a^ Intrinsic capacity is defined by the World Health Organization as the combination of the individual’s physical and mental (including psychosocial) capacities.

## Synergies with existing approaches

Numerous frameworks for organizing care for older people have been developed and are used in different parts of the world.^33^^,^^34^ In addition, chronic care models, which share features with models designed for older people, have also been developed and implemented internationally.^35^^–^^38^ Although a full review of these models is beyond the scope of this paper, they have many features in common with WHO’s proposed approach to integrated health care for older people, including: (i) standardized assessments and care plans shared among all providers; (ii) shared decision-making and goal-setting; (iii) support for self-management; (iv) multidisciplinary teams; (v) unified information or data-sharing systems; (vi) community linkages or integration; and (vii) supportive leadership, governance and financing mechanisms.

Some small differences between models exist, which probably resulted from the different contexts in which they were developed or from variations in their intended applications. For example, there are differences in the models’ target outcomes, in the position and prominence of social services in the model and in the relative emphasis placed on macro-, meso- and micro-level actions. Crucially, WHO’s approach places the older person at the centre of service delivery; other models may take into account families, health-care workers and community members.^36^^,^^38^ In addition, WHO’s approach gives prominence to integration at the level of clinical care, though action at the macro level, involving elements of the health system, is also important.

Overall, WHO’s approach to integrated health care for older people is consistent with WHO’s more general framework on integrated, people-centred health services, which was adopted by the Sixty-ninth World Health Assembly in 2016.^39^ The framework calls for a change in the way health services are funded, managed and delivered and proposes five interdependent strategies that must be implemented to enable health services to become more people-centred and integrated: (i) engaging and empowering people and communities; (ii) strengthening governance and accountability; (iii) reorienting the model of care; (iv) coordinating services within and across sectors; and (v) creating an enabling environment.^40^ In particular, WHO’s guidance on integrated health care for older people meets the requirement to base service priorities on an individual’s needs throughout their life course, as expressed in strategy (iii) of WHO’s framework on integrated, people-centred health services.^40^

## Integrated care for older people in practice

Documented examples of integrated care for older people are scarce, particularly from low- and middle-income countries. Several examples were noted in the *World report on ageing and health*,^1^ but most focused on a specific aspect of older people’s health or of the care system and did not adopt a truly comprehensive approach to changing health care for older people.

Moreover, it is difficult to assess or compare different programmes because little direct evidence is available on the way integrated health care was provided for older people or on its extent. Often terminology varies between programmes, as does the range of factors that should be included in, or excluded from, analyses. One key issue is the distinction between integrated care (which can be considered as including social care) and integrated health care. The information currently available is derived mainly from case studies, which exhibit large variations in the way integrated care was implemented in practice and in how programmes were evaluated. For example, the European Commission published a guide to innovative healthy ageing programmes being implemented in more than 30 regions, cities and communities in Europe – many featured the reform of some aspect of integrated care but descriptions of the programmes varied widely.^41^ Only a few structured, cross-case analyses have been carried out.

Exemplar national programmes do exist but they tend to have been implemented at a subnational, governmental level or in a small geographical area.^1^ A few countries, such as Singapore and Scotland in the United Kingdom of Great Britain and Northern Ireland,^42^ have made substantial improvements in care for older people at the national level. However, programmes in most countries are characterized by a more ad hoc approach: integrated care may be delivered in a municipality or a subnational region but not yet have taken hold across the country. Two studies on seven subnational integrated care programmes for older people, all in high-income countries (examples in Table 1) found a range of different types of integration. The types ranged from fully-integrated health and social care to approaches that built alliances to coordinate care – these alliances were often based on contractual relationships between otherwise separate partners.^42^^,^^45^ Additionally, numerous countries and organizations are working on integrated care delivery models that can cope with the challenges presented by the increasing prevalence of chronic diseases and multimorbidity.^46^^–^^48^ Although these models do not specifically target older people, their underlying principles are similar to those of WHO’s approach to integrated health care for older people.

**Table 1:** Experience with integrated health care for older people, worldwide, 1999–2016

Country and programme	Principle features of integrated care programme^a^	Results
Australia: coordinated care trials^42^^,^^43^	(i) Whole population approach, which encompassed improvements in access to, and in the delivery of, primary health-care services and in care coordination within the community; (ii) care coordination for people with chronic and complex needs; (iii) information management and technology; and (iv) the creation of robust mechanisms to resolve conflicts.	(i) Clients felt supported and less anxious and general practitioners were very satisfied; (ii) fewer emergency department visits and shorter hospital stays; and (iii) fewer referrals to community health services.
Brazil: Ageing in the National Family Health Programme (case study)^b^	(i) Home visits undertaken by a multidisciplinary team comprising a doctor, nurse and social worker; (ii) health workers were trained to assess frailty and functioning; and (iii) strong referral links to primary health-care clinics were established.	(i) Results have not yet been documented.
Canada (Province of Quebec): Program of Research to Integrate Services for the Maintenance of Autonomy (PRISMA)^44^	(i) Coordination between decision-makers and managers; (ii) single entry point to care; (iii) case management; (iv) individualized service plans; (v) single assessments; (vi) focus on clients’ functional autonomy; and (vii) computerized clinical chart for communicating between institutions on client monitoring.	(i) Increased client satisfaction and empowerment; (ii) lower incidence of functional decline; (iii) fewer unmet needs; (iv) fewer emergency department visits and hospitalizations; (v) no increase in consultations with health professionals or in the need for home care services; and (vi) better system performance at no additional cost.
Thailand: “Friends Help Friends” project (case study)^c^	(i) Long-term care lead by the health ministry; (ii) support for informal carers who are providing long-term care; (iii) informal carers and community volunteers are formally engaged in the system and carry out home visits and functional assessments; and (iv) a health professional linked to the nearby health centre provides supervision and logistic support.	(i) Results have not yet been documented.
United Kingdom (England): case study programmes^42^	(i) Real integration involving vertical integration (i.e. hospital to home) and horizontal integration (i.e. multidisciplinary teams); (ii) people in the community with complex needs targeted; (iii) multidisciplinary teams comprising care coordinators, community nurses, occupational therapists, physiotherapists and social workers; and (iv) funds from National Health Service clinical commissioning group and local authority are pooled.	(i) Increased staff motivation and positive evaluations from general practitioners; (ii) shorter waiting times before receiving long-term care support; (iii) fewer emergency admissions; (iv) fewer bed days and shorter hospital stays; (v) fewer residential home placements; and (vi) better system performance at no additional cost.

^a^ Information was obtained from the *World report on ageing and health*.^1^

^b ^Eduardo Augusto Duque Bezerra, Pernambuco State Health Secretary, Personal communication, 2015.

^c^Ekachai Piensriwatchara and Puangpen Chanprasert, Department of Health, Ministry of Public Health, Thailand, Personal communication, 2015.

## Future challenges

Several factors appear to be limiting the widespread adoption of integrated care for older people, including a lack of political commitment, gaps in general knowledge about integrated care, problems with implementation and inadequate sharing of experiences with integrated care internationally.

The inherent complexity of organizing and delivering coordinated care for a large older population can be discouraging and can undermine efforts to introduce and scale up programmes. Moreover, as older people vary considerably in their level of intrinsic capacity, a wide range of clinical interventions must be made available and appropriately targeted. Further, the older person’s capacity can change rapidly, which means that the care system must be able to respond quickly to changes in need with updated care plans and services. In addition, any reform of the care system designed to provide truly integrated, person-centred care for older people must involve a range of different organizations and providers, including both health care and social care providers and providers in sectors such as transportation. Political and administrative leaders might look at the scale of the task and conclude it requires too great an effort to warrant action.

A further complication is the lack of evidence showing that integrated care for older people can produce cost savings. To date, studies have demonstrated that coordinated care is cost-neutral – integrated, person-centred care can be delivered for roughly the same cost as standard care.^49^ The lack of a demonstrable impact on cost might make decision-makers even more reluctant to consider integrated care. However, it is important to note that, although an initial investment (e.g. for training and additional personnel) is necessary, over the medium to long term, cost savings are to be expected, due, for example, to less duplication and better coordination of services. Integrated care should be regarded as a lengthy journey that necessitates a long-term perspective.

Another challenge is inadequate sharing of knowledge between programmes and countries. At the global level, WHO and other bodies have started to collate information on outcome indicators as part of efforts to establish a measurement basis for WHO’s framework on integrated, people-centred health services.^39^ However, knowledge about the implementation and spread of initiatives in the field of ageing does not flow freely. This is partly due to a lack of consensus on the definitions of widely used concepts, such as integrated care, person-centred care and people-centred care, and a lack of measurement tools. Consequently, there are no clear criteria or measures for determining whether, and to what extent, integrated care for older people is actually being delivered in any particular setting.

There is also a lack of consensus on what constitutes a positive outcome for older people. Traditionally, health-care research has used indicators of disease, disability, longevity, patient and provider satisfaction, health-care utilization, hospitalization, institutionalization and cost. In contrast, the main aim of integrated care for older people is not to manage disease or prolong life but is, instead, to optimize older people’s intrinsic capacity over their life course and, hence, ensure healthy ageing. A different set of outcome indicators is needed – indicators that reflect intrinsic capacity, functional ability, quality of life and the attainment of goals defined by the older person. Some indicators already exist but others have still to be developed. In addition, it is crucial that any measurement tools used with older people are validated in low- and middle-income countries.

Finally, only a few easily accessible, policy guidance and implementation tools for countries at different levels of development exist. Policy guidance documents and implementation tools have been developed mainly in high-income countries and their applicability to other countries and regions, and to low- and middle-income countries in particular, remains unclear.

In general, the focus of clinical care for older people has to undergo a fundamental shift. This does not mean that existing organizational structures must merge but, rather, that a wide array of service providers must work together in a coordinated way. Experience to date indicates that most programmes designed to provide integrated care for older people have taken a bottom-up approach to change and have been supported by higher-level policy and by mechanisms for sharing financing and accountability within teams. Nevertheless, few evaluations of these programmes have been done and little attention has been paid to how they might be adapted for low-resource settings. Moreover, the lack of a consensus on definitions and outcome indicators makes it even more difficult to draw firm conclusions from past experience.

In conclusion, achieving the goals of WHO’s global strategy and plan of action on ageing and health^2^ requires political commitment to integrated health care for older people, the development of coherent health systems policy, and normative guidance on the implementation and evaluation of integrated care both nationally and internationally.
